# Fast field echo resembling a CT using restricted echo-spacing (FRACTURE) sequence for shoulder joint in normal dogs

**DOI:** 10.3389/fvets.2024.1298133

**Published:** 2024-01-30

**Authors:** Sanghwa Ryu, Soomin Park, Eunjee Kim, Hyeonjae Woo, Chang-yeop Jeon, Junghee Yoon, Jihye Choi

**Affiliations:** ^1^Department of Veterinary Medical Imaging, College of Veterinary Medicine, Seoul National University, Seoul, Republic of Korea; ^2^National Primate Research Center, Korea Research Institute of Bioscience and Biotechnology, Cheongju, Republic of Korea

**Keywords:** 3.0-T, 3D gradient echo sequence, canine, magnetic resonance imaging, shoulder

## Abstract

Shoulder disease is a common cause of forelimb lameness in dogs. Determining the precise underlying cause of shoulder lameness can be challenging, especially in veterinary practice. Computerized tomography (CT) is often the preferred imaging modality for bone evaluation; however, it uses ionizing radiation and provides limited soft tissue contrast. Conversely, magnetic resonance imaging (MRI) offers excellent soft tissue contrast but has limitations in bone imaging. This study aimed to introduce a new technical innovation that enhances cortical and trabecular bone contrast on MRI, which we refer to as Fast Field Echo Resembling a CT Using Restricted Echo-Spacing (FRACTURE). In this prospective pilot study, we aimed to evaluate the use of FRACTURE, CT, and conventional MRI sequences in assessing the normal canine shoulder using a 3.0 Tesla MRI scanner. Five research beagle dogs were included, and the following pulse sequences were acquired for each dog (1): three-dimensional (3D) FRACTURE, (2) T2-weighted (T2W) images using 3D turbo spin echo (TSE), (3) T1-weighted (T1W) images using 3D TSE, (4) PD-weighted (PDW) images using 3D TSE, and (5) CT. Various parameters, including the delineation of cortical bone (intertubercular groove, greater tubercle, and lesser tubercle), conspicuity of the trabecular bone, shoulder joint visualization, and image quality, were measured for each dog and sequence. In all sequences, the shoulder joint was successfully visualized in all planes with mild motion artifacts. The intertubercular groove was best visualized on CT and FRACTURE. Both the greater and lesser tubercles were easily identified on the CT, FRACTURE, and PDW images. The trabecular pattern scored significantly higher in the CT and FRACTURE images compared to the T1W, T2W, and PDW images. Overall, the visualization of the shoulder joint was excellent in all sequences except for T1W. The use of FRACTURE in combination with conventional MRI sequences holds promise for facilitating not only soft tissue evaluation but also cortical and trabecular bone assessment. The findings from this study in normal dogs can serve as a foundation for further FRACTURE studies in dogs with shoulder diseases.

## 1 Introduction

The shoulder joint in dogs is characterized by its complexity, consisting of various passive and active soft tissue structures responsible for stabilization. Traumatic or degenerative injuries of the shoulder, including bone disorders such as osteochondrosis or osteochondritis dissecans (OCD), as well as soft tissue disorders such as supraspinatus tendinopathy, biceps tendinopathy, infraspinatus muscle contracture, other tendinous and ligament injuries, and adhesive capsulitis, can lead to chronic shoulder pain and lameness in dogs (1–3). Many shoulder joint diseases, originally generated from soft tissue, commonly induce secondary bone alterations and vice versa. In canine shoulder OCD, the displaced joint mice can irritate the surrounding biceps brachii tendon and result in tendinopathy (4). In contrast, shoulder tendinitis/tenosynovitis may result in an avulsion fracture at the proximal insertion of the biceps brachii. Consequently, a comprehensive assessment including secondary bone and soft tissue changes becomes crucial for the acute diagnosis of shoulder joint diseases.

Determining the precise underlying cause of shoulder lameness in dogs can be challenging. Radiography is commonly used for screening the shoulder in dogs showing forelimb lameness; however, most conditions can be within normal radiographic findings due to the soft tissue nature of the injuries involving the tendon, ligament, or cartilage. Ultrasonography can be used for examining soft tissues, in contrast to radiography. However, the reliability of an ultrasonographic diagnosis is highly dependent on the operator and requires the operator’s experience and a comprehensive understanding of the anatomy of the shoulder. Arthroscopy is also used for soft tissue evaluation by the direct visualization of intra-articular abnormalities but is hindered by its invasive nature.

Advanced modalities such as CT and MRI allow the delineation of the anatomic changes in the shoulder by providing trans-sectional images and enhancing the image resolution in veterinary practice (5). CT can provide details of the bone surface, allow the determination of calcification and fragmented structures, and diagnose fractures and dislocations in shoulder joint diseases effectively. On the CT images of the shoulder, new bone formation can be identified within the insertion of the tendon and an avulsion fracture in the tendon rupture/tear. This highlights the importance of evaluating the morphology and changes in the ligament or tendon attachment sites, as well as the presence of fractures and mineralization in bone, through CT imaging.

Meanwhile, MRI is considered essential because the prevalence of soft tissue disorders, including ligaments and tendons is high in the shoulder. MRI can provide superior soft tissue resolution, global assessment, and multiplanar imaging capabilities, and it is non-invasive and non-ionizing (6–8). However, many parts of the shoulder, such as the cortical bone, tendons, ligaments, and periosteum, primarily comprise tissues with tightly bound protons that decay extremely quickly (9). This rapid decay results in extremely short T2 and approximate mean T2 relaxation times of the cortical bone (0.4–0.5 ms), ligament (4–10 ms), tendon (4–7 ms), and periosteum (5–11 ms) (10).

Various imaging techniques, including ultrashort echo time (UTE), zero echo time (ZTE), and fast field echo resembling a CT using restricted echo-spacing (FRACTURE), are being researched in MRI to visualize bone with extremely short T2 decay. UTE and ZTE sequences, both based on fast gradient echo sequences, shorten the TE by less than 1 ms and can depict the signal from the short T2 components in tissues rapidly before it decays and visualize them as hyperintense signals (9). Both sequences are useful for detecting tissues with extremely short T2 decay; however, their availabilities are limited to academic environments because they require specific hardware and software components, including fast transmit-receive switching, precise radiofrequency waveform transmission, high gradient performance, and gradient calibration. The recently proposed FRACTURE is based on a high-resolution 3D gradient echo pulse sequence that offers a similar contrast to CT through the use of constant echo-spacing and post-processing subtraction (11). It reduces the contrast of surrounding tissues and creates a high contrast between the surrounding tissues and bones by optimizing the low flip angle, echo time, and repetition time settings. This technique provides a low-signal bone contour compared to the high-signal fatty bone marrow and soft tissues and results in high bone contrast. The powerful advantages of FRACTURE are its ability to be used on conventional MRI scanners as well as its ability to generate high spatial resolution images. Moreover, it can achieve a lower specific absorption rate by utilizing smaller flip angles and reducing the absorption of electromagnetic waves in the body. By using FRACTURE pulse sequences, the acquisition of CT-like MR imaging has been applied to human medicine.

However, to the best of our knowledge, the application of FRACTURE to any of the joints has not yet been reported in animal studies. Considering the tightly bound proton components in the shoulder and the prevalence of the diseases in the region, FRACTURE application to the shoulder joints may increase the sensitivity of diagnosis and efficacy by solving the long anesthesia time and cost due to taking both MRI and CT scans for assessing the bone and soft tissue in shoulder diseases. In this study, FRACTURE was performed in the shoulder joint and compared with CT images in normal dogs. We hypothesized that FRACTURE would visualize the cortical bone and trabecular bone of the shoulder joint better than conventional MR sequences and have substantial agreement with CT images. This study aimed to evaluate the feasibility of FRACTURE MR imaging to assess the osseous part of the shoulder joint and describe the FRACTURE findings of the shoulder region in healthy dogs. We hypothesized that the osseous visuality of the shoulder joint region on the FRACTURE sequence would be significantly correlated with that on CT. This correlation could be used as a reliable MRI sequence for assessing the cortical bone in the shoulder joint.

## 2 Materials and methods

This study was approved by the Institutional Animal Care and Use Committee at Seoul National University, and the dogs were cared for according to the Guidelines for Animal Experiments of Seoul National University (SNU IACUC-230726-1).

### 2.1 Animal

In this method-comparison, prospective, pilot study, five adult purpose-bred beagle dogs (three male and two female dogs) were used. The median age was 3 years (2–5 years), and the median weight was 13 (9.9–15.5) kg. All dogs were clinically healthy, based on a physical examination, blood pressure measurement, complete blood count, and serum biochemistry, including alkaline phosphatase, aspartate aminotransferase, alanine aminotransferase, blood urea nitrogen, creatinine, glucose, total bilirubin, albumin, total protein, globulin, Υ-glutamyl transpeptidase, calcium, inorganic phosphorus, electrolyte concentration, radiographs, and ultrasonography. The dogs had no history of lameness. The right lateral and ventrodorsal radiographs of the shoulder were obtained using a digital radiographic system (EVA-HF525, Gemss-Medical, South Korea; maximum tube voltage 125 KV, maximum tube current 500 mA) with a cesium–iodine-based flat panel detector (FDX4343R, Gemss-Medical, South Korea) integrated into the table. The radiography was obtained by placing the dog directly on the table with the detector below, without the use of a grid. A focal film distance of 100 cm was used, with exposure factors ranging from 50 to 70 kV and 3 mAs.

### 2.2 Anesthesia and schedule

In each dog, CT and MRI of the shoulder were performed randomly at a minimum of 7-day intervals. After 8 h of fasting, a 24-gauge catheter was aseptically placed into the saphenous vein. Anesthesia was induced with an intravenous injection of 10–15 μg/kg of medetomidine hydrochloride (Domitor, Zoetis, Finland) and 2 mg/kg of alfaxalone (Alfaxan, Jurox Pty Ltd., Australia). Following endotracheal intubation, anesthesia was maintained with isoflurane (2–4%; Ifran, Hana Pharm) delivered in 100% oxygen (1–2 L/min). During the induction and maintenance of anesthesia, heart rate and oxygen saturation were continuously monitored using a respiratory sensor and ECG tracing, respectively. Following the MRI and CT scans, the overall condition of each dog and any anesthesia-related side effects, including vomiting, depression, and anorexia, were monitored for 5 days.

### 2.3 MRI examinations

**Figure 1:**
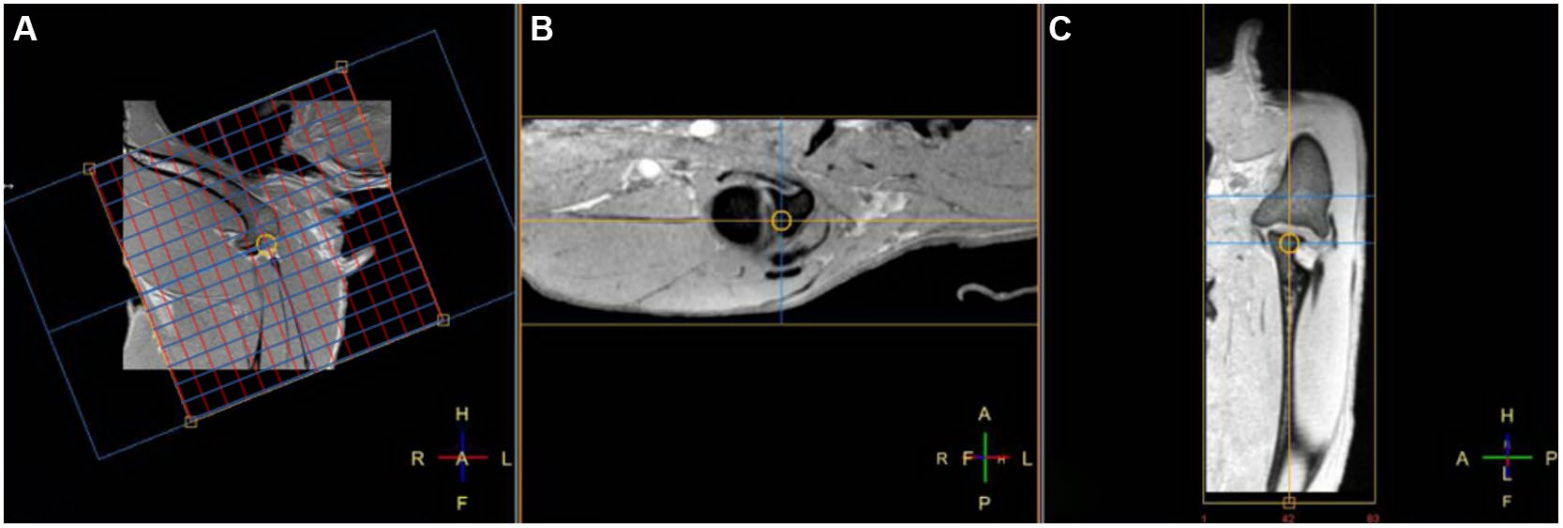
Fast field echo resembling a CT echo-spacing (FRACTURE) MR images of a right shoulder in the sagittal **(A)** TE, 2.3 ms; TR, 16 ms and reconstructed transverse **(B)** and dorsal **(C)** planes on a 3D fast field echo acquisition sequence. In the sagittal plane **(A)**, the red line represents the guideline for the dorsal plane, and the blue line represents the guideline for the transverse plane, respectively.

In right lateral recumbency, the right shoulder was placed downward and extended with a 130–150° angle, and the left shoulder was placed upward, parallel to the right shoulder joint. Then, a 32-channel cardiac coil was placed around both shoulder joints, and MRI images were obtained using a 3.0-T MRI (Philips Achieva, Philips Healthcare, Best, Netherlands). At first, three orthogonal planes with T1-weighted (T1W) images using 3D turbo field echo scans were obtained as a localizer; transverse images were obtained perpendicular to the long axis of the spine of the scapula, and the dorsal and sagittal images were made parallel to the long axis of the spine of the scapula and humerus. Then, 3D image acquisition of T1W, T2-weighted (T2W), and proton density-weighted (PDW) using turbo spin-echo (TSE) and FRACTURE imaging in the sagittal plane was performed, and transverse, sagittal, and dorsal planes of the right shoulder were obtained in each sequence. Acquisition sequences were used, and then the dorsal and transverse planes were reconstructed (Figure 1). The total scan duration and image acquisition time for each sequence were measured automatically. The parameters used for each sequence are shown in Table 1.

**Table 1:** MRI sequence parameters used for the shoulder joints in dogs.

Parameter	T1W (TSE)	T2W (TSE)	PDW (TSE)	FRACTURE (FFE)
TR (ms)	400	1,300	1,000	16
TE (ms)	110	144	32	2.3
FA (degree)	90	90	90	12
Echoes	1	1	1	5
Turbo factor	20	50	40	–
NEX	1	1	1	1
FOV (mm)	160 × 160 × 60	160 × 160 × 60	160 × 160 × 60	160 × 160 × 60
Matrix	268 × 266	268 × 266	268 × 266	268 × 266
Voxel size (mm)	0.6 × 0.6 × 0.6	0.6 × 0.6 × 0.6	0.6 × 0.6 × 0.6	0.6 × 0.6 × 0.6
Image acquisition time (min:sec)	16:53.2	21:56.9	21:06.0	07:01.2
Plane	Sagittal			

TR, repetition time; TE, echo time; FA, flip angle; ST, slice thickness; NEX, number of excitations; FOV, field of view; T2W, T2-weighted; T1W, T1-weighted; TSE, turbo spin echo; 3D, three dimensional; FFE, fast field echo.

### 2.4 CT examinations

After placing the dog in dorsoventral recumbency with the forelimbs extended cranially, while maintaining an extension angle of 130–150° between the spine of the scapula and the long axis of the humerus, a CT scan of the shoulder was performed using a 160-slice helical CT scanner (Aquilion Lightning 160 MODEL TSX-036A, Canon Medical System, Otawara, Japan). Lateral and dorsoventral scout views were taken to define the scan field of view from the mid-scapula to the mid-humerus regions. CT images of the right shoulder joint were obtained with the following settings: a tube voltage of 120 kVp, a tube current of 200 mA, a slice thickness of 0.5 mm, and 1.0 s of tube rotation time. The image matrix size was 512 × 512 mm^2^, with the field of view being 300 × 300 mm^2^. All CT images were reconstructed into transverse, sagittal, and dorsal planes with a thickness of 0.6 mm slice interval of 0.6 mm, and a bone algorithm. Utilizing the reconstructed sagittal plane, reformatted CT images were acquired to correspond to MRI scans.

### 2.5 Image analyses and comparison of MRI and CT

MRI and CT images of the shoulder were sent to a workstation and assessed by two observers (S.H.R and S.M.P) each having 2 years of radiology experience, separately in a blinded manner. CT images were evaluated using a bone window (window width of 2000 HU and a window level of 500 HU).

In qualitative assessment, the delineation of the cortical bone, the conspicuity of the trabecular bone, the conspicuity of the shoulder joint, and image quality were evaluated (Table 2). The qualitative assessment was performed, which was selected from each plan in each MRI sequence and compared with the CT images at the same level by scrolling the image slices. The delineation of the cortical bone, conspicuity of the trabecular bone, and conspicuity of the shoulder joint were scored using a 4-point scale: grade 1 = not visible at all; grade 2 = barely visible, but detectable and identified by its location and contrast; grade 3 = visible, but a little burring; and grade 4 = easily visible with a clear margin. If there are two or more planes that need to be evaluated, each plane is scored and then averaged. Image quality was assessed about the degree of blurring, partial volume, motion artifact, magic angle artifact, beam-hardening artifact on CT, and the amount of noise and scored using a 4-point scale (12): grade 1 = impossible to interpret images due to marked artifacts; grade 2 = possible to interpret images despite artifacts; grade 3 = artifact is present but minimal, not affecting images; and grade 4 = optimal image quality without artifact.

**Table 2:** Evaluation factors, sites, and planes for qualitative assessment.

Evaluation factors	Evaluated sites	Planes and regions
Delineation of the cortical bone	Intertubercular groove where the biceps brachii tendon is well visualized	Sagittal plane; between the well-visualized segments of the humeral head and the greater tubercle of the humerus. Transverse plane; between the well-visualized portions of the humeral body and the acromion of the scapula.
	Greater tubercle where the supraspinatus and infraspinatus tendon are well visualized	Sagittal plane; between the well-visualized segments of the humeral head and the greater tubercle of the humerus. Transverse plane; between the well-visualized portions of the humeral body and the acromion of the scapula.
	Lesser tubercle where the subscapularis tendon is well visualized	Dorsal plane; between the visualization of the spine of the scapula and the well-defined humeral diaphysis segments.
Conspicuity of the trabecular bone	Humerus epiphysis	Sagittal, dorsal, and transverse planes
Conspicuity of the shoulder joint	Shoulder joint	Sagittal plane
Image quality	Whole images	Sagittal, dorsal, and transverse planes

### 2.6 Statistical analyses

Statistical analyses were performed by one statistician (J.R.P) using commercially available software (SPSS Statistics, Version 27 for Windows, IBM Corp., Chicago, IL, USA). A Friedman test with a *post-hoc* Wilcoxon signed-rank test was used for analyzing the difference in the definition of the cortical bone, conspicuity of the trabecular bone, and conspicuity of the shoulder joint among the MR sequences and CT images (13). Agreement between two observers for the MRI and CT images was evaluated using the interclass correlation coefficient (ICC) and 95% confidence intervals for the datasets in both parts of the study. The correlation was considered poor, fair, good, and excellent if the ICC was <0.4, 0.41–0.6, 0.61–0.8, and > 0.8, respectively (14). Data are presented as mean ± standard deviation (SD). The level of significance was set at *p* < 0.05.

## 3 Results

### 3.1 MR imaging and features of the shoulder joint

MR images of the right shoulder joint were obtained successfully using all MRI sequences and CT in all dogs without any complications, including hypothermia, abnormal heart rate, hypotension, and difficult recovery. MRI scans were completed in approximately 67 min after initiating image acquisition for each dog.

**Figure 2:**
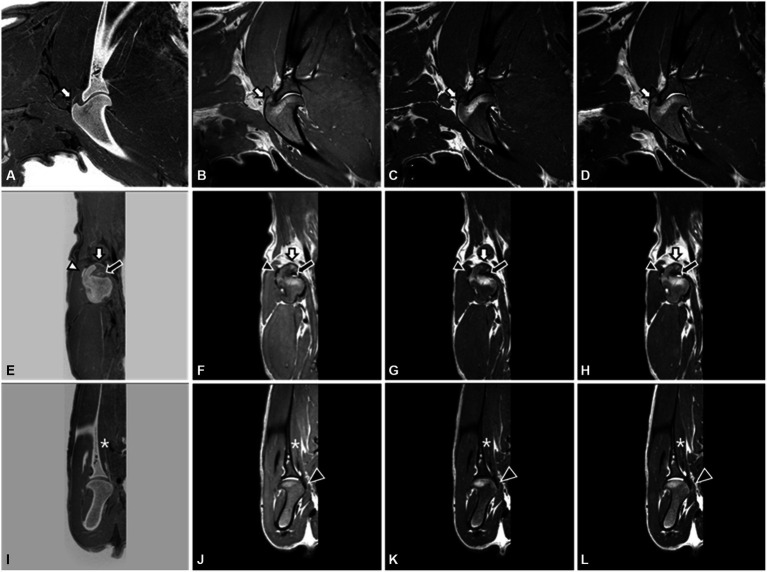
3.0T Magnetic resonance images of the right shoulder joint in a dog. FRACTURE **(A,E,I)**, PDW **(B,F,J)**, T1W **(C,G,K)**, and T2W **(D,H,L)** images using 3D FSE. Sagittal image **(A–D)** and transverse image **(E–H)** at the level of the supraspinatus tendon (white arrow) insertion on the grater tubercle. The biceps brachii tendon (black arrow) and infraspinatus tendon (white arrowhead) are well visualized on the transverse plane. On the dorsal images **(I–L)**, the subscapularis muscle (asterisk) and tendon (black arrowhead) are well identified.

MRI images showed the tendons and muscles around the shoulder joint, such as the biceps brachii tendon, supraspinatus tendon, infraspinatus tendon, subscapularis muscle, and tendon, compared with CT images (Figure 2). The biceps brachii tendon and its sheath were directly visualized on sagittal and transverse T2W, T1W, and PDW images. The tendon appeared uniformly hypointense on all planes. The supraspinatus tendon was visualized well in sagittal and transverse sequences. Within the muscle belly, the proximal segment of the tendon showed a slight isointensity compared to the surrounding muscles. However, as it is inserted broadly onto the cranial aspect of the greater tubercle of the humerus, there is a consistent hyperintense signal observed on T2W, T1W, and PDW images. The infraspinatus tendon was visualized as distinct and hypointense, with a clear and well-defined structure in all planes and sequences. It was flattened in the mediolateral orientation and gradually increased in thickness as it extended distally and ultimately inserted onto the lateral margin of the greater tubercle of the humerus. The subscapularis muscle and tendon were visualized well in the transverse and dorsal planes, regardless of MRI sequences. The muscle runs along the medial aspect of the scapula, and the tendon inserts onto the lesser tubercle of the humerus, positioned immediately distal to the joint. In the FRACTURE sequence, visualizing the infraspinatus tendon and subscapularis muscle was straightforward, and identifying the supraspinatus tendon and biceps brachii tendon was feasible. The subscapularis tendon was not confirmed in a 3D MRI. In the FRACTURE sequence, all tendons exhibited hypersignality when compared to conventional MRI sequences because of black-and-white inverted imaging in FRACTURE.

Table 3 presents the scores of the qualitative assessment of CT and MRI images, which did not show normality. Generally, the qualitative assessments showed a strong agreement between the two observers, except in the evaluation of the conspicuity of the trabecular bone. However, this discrepancy might be attributed to the limited sample size, even though all scores were above 3 points (Tables 4, 5).

**Table 3:** Qualitative assessments of the right shoulder joint on magnetic resonance imaging and computed tomography in healthy dogs.

Evaluation factors	Evaluation sites	CT	T1W	T2W	PDW	FRACTURE
Cortical delineation	Intertubercular groove	4 ± 0.00^a^	1.9 ± 0.73^b^	2.8 ± 0.63^c^	2.8 ± 0.63^c^	3.4 ± 0.51^d^
	Greater tubercle	4 ± 0.00^a^	1.9 ± 0.73^b^	2.9 ± 0.56^c^	3.0 ± 0.66^c^	3.6 ± 0.51^d^
	Lesser tubercle	4 ± 0.00^a^	2.2 ± 0.44^b,d^	2.8 ± 0.44^c,d^	3.2 ± 0.83^a,b,c^	3.8 ± 0.44^a^
Conspicuity of the trabecular bone	Humerus epiphysis	3.33 ± 0.48^a^	1.67 ± 0.72^b,c^	1.8 ± 0.67^c^	1.53 ± 0.74^b^	3.27 ± 0.70^a^
Conspicuity of the shoulder joint	Shoulder joint	4 ± 0.00^a^	3.6 ± 0.54^b^	2.4 ± 0.54^a^	4 ± 0.00^a^	4 ± 0.00^a^
Image quality	Whole images	2.6 ± 0.54^a^	3.0 ± 0.70^a,b^	3.6 ± 0.54^b^	3.6 ± 0.54^b^	2.8 ± 0.44^a^

All data are presented as means ± standard deviations.

^a–d^Within a row, values with superscripted letters differ using a Wilcoxon signed-rank test (*p* < 0.05).

CT, computed tomography; T1W, T1-weighted image; T2W, T2-weighted image; PDW, PD-weighted image; FRACTURE, Fast field echo resembling a CT using restricted echo-spacing echo.

**Table 4:** Interclass correlation coefficient values for interobserver reliability of the qualitative assessments of MR images of canine shoulder joints.

Evaluation factors	Evaluation sites	ICC	95% confidence interval
Cortical delineation	Intertubercular groove	0.89	0.73–0.95
	Greater tubercle	0.84	0.60–0.93
	Lesser tubercle	0.84	0.61–0.93
Conspicuity of the trabecular bone	Humerus epiphysis	0.97	0.93–0.99
Conspicuity of the shoulder joint	Shoulder joint	0.91	0.77–0.96
Image quality	Whole images	0.95	0.88–0.98

**Table 5:** Interclass correlation coefficient values for interobserver reliability of the qualitative assessments of CT images of canine shoulder joints.

Evaluation factors	Evaluation sites	ICC	95% confidence interval
Cortical delineation	Intertubercular groove	1	0
	Greater tubercle	1	0
	Lesser tubercle	1	0
Conspicuity of the trabecular bone	Humerus epiphysis	0.54	−3.41–0.95
Conspicuity of the shoulder joint	Shoulder joint	1	0
Image quality	Whole images	0.87	−0.25–0.98

### 3.2 Delineation of the cortical bone

On CT scans, the outlines of the intertubercular groove, greater tubercle, and lesser tubercle are quite clear across all planes.

FRACTURE showed a clearer definition of the cortical bone in the intertubercular groove and greater tubercle, showing significantly higher scores (3.4 and 3.6, respectively) than those of T1W, T2W, and PDW. There was a significant difference between FRACTURE and T1W, T2W, and PDW (*p* < 0.05). In particular, the identification of the intertubercular groove and greater tubercle was a challenge in the T1 sagittal plane, while in the transverse plane, they were barely visible in most dogs.

A significant difference was detected between FRACTURE and CT on the intertubercular groove and greater tubercle, with a confirmed value of *p* of 0.014 and 0.046, respectively. Clear visibility is observed in both the sagittal and transverse planes in CT. However, in the FRACTURE sequence, the transverse plane exhibits better visibility compared to the sagittal plane. Following CT and FRACTURE, PDW imaging provided the third-best visualization of the greater tubercle. Cortical delineation is easily visualized in both the sagittal and transverse planes in the PDW sequence. Between PDW and the two sequences, a significant difference was observed with a value of *p* of less than 0.05.

**Figure 3:**
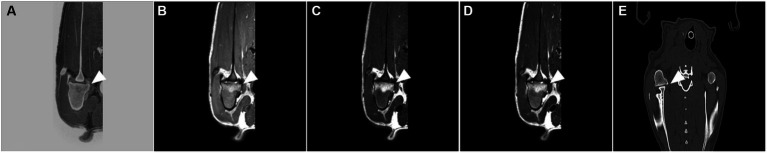
FRACTURE **(A)**, PDW **(B)**, T1W **(C)**, T2W **(D)**, and CT **(E)** images of the right shoulder in the dorsal plane in a dog. The lesser tubercle (white arrow) to which the subscapularis attaches is clear in the order of FRACTURE **(A)**, PDW **(B)**, and computed tomography **(E)**.

The definition of the lesser tubercle, to which the subscapularis attaches, was clear in the order of CT, FRACTURE, and PDW. In contrast to the intertubercular groove and greater tubercle, the lesser tubercle shows no significant difference between these sequences (*p* > 0.05). In contrast to T1W and T2W, where the lesser tubercle was barely visible, in CT, FRACTURE, and PDW, the cortical delineation of the lesser tubercle could be clearly distinguished from the surrounding muscles and ligaments (Figure 3).

### 3.3 Conspicuity of the trabecular bone

**Figure 4:**
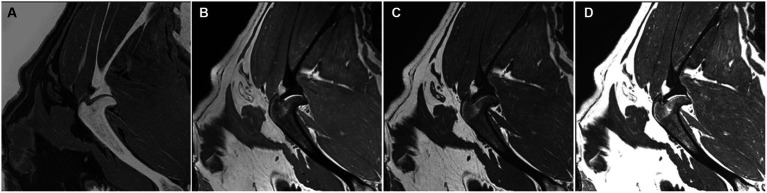
FRACTURE **(A)**, PDW **(B)**, T1W **(C)**, and T2W **(D)** images of the right shoulder in the sagittal plane in a dog. The trabecular pattern in the epiphyseal region of the humerus appears indistinguishable and manifests as a coalescence of elevated signal intensity.

The trabecular pattern of the bones was observed in detail in the CT and FRACTURE images, except for one dog (Figure 4). There was a coalescence of high-signal intensities within the cancellous bone in the T1W, T2W, and PDW.

The mean score of the conspicuity of the trabecular pattern was significantly higher in the CT and FRACTURE images compared to conventional MRI sequences such as T1W, T2W, and PDW, and there was no significant difference between FRACTURE and CT for the visualization of the trabecular pattern (*p* = 0.705).

### 3.4 Conspicuity of the shoulder joint

The shoulder joint was visualized clearly in CT and all MR images except for T1W. Specifically, CT, FRACTURE, and PDW consistently achieved a perfect score of 4 points for all dogs. Both CT and FRACTURE displayed a clear boundary between the glenoid cavity and the head of the humerus. In T2W and PDW images, cortical delineation of the glenoid cavity and head of the humerus, along with a hypersignal joint space, was observed, resulting in increased contrast. Both FRACTURE and PDW were found to be non-inferior to conventional CT in terms of shoulder joint visualization (*p* = 1.00). Additionally, T2W exhibited similar joint visualization compared to CT and FRACTURE (*p* = 0.157).

### 3.5 Image quality and artifact

Artifacts were observed in most MR sequences, including partial volume and motion artifacts. The magic angle artifact was not observed in any images. The image quality of the T2W and PDW sequences was superior to that of the FRACTURE sequence, which showed more motion artifacts (Table 4). However, image quality was sufficient to allow image interpretation in all MR images with more than grade 2, except for T1 images in one dog, which showed grade 1. In CT images, beam-hardening artifacts were observed due to the angle of the humerus. Consequently, the mean scores of image quality for CT and FRACTURE were below 3.0.

## 4 Discussion

In this study, the FRACTURE sequence was applied to assess the shoulder joint and compared with CT images and conventional MRI sequences in normal dogs. FRACTURE sequences were feasible for the acquisition of MR images that look similar to CT of the shoulder in dogs. FRACTURE exhibited superior contrast for both cortical and trabecular bone compared to conventional MRI sequences.

The quality assessment of the definition of the cortical bone was highest in CT images in the intertubercular groove and greater tubercle compared to FRACTURE and the conventional MR sequences. However, FRACTURE offered clearer cortical delineation than the other T1W, T2W, and PDW sequences.

The cortical delineation of the intertubercular groove, where the biceps brachii tendon courses distally (15), was notably higher in CT and FRACTURE than conventional MRI sequences. Meanwhile, in most transverse planes, the intertubercular groove was easily visible in PDW sequences. Because transverse images are perpendicular to the long axis of the humerus, they provide better visibility of cortical delineation (8). In particular, with PDW, the contrast in proton density between bone and surrounding muscle makes cortical delineation easily visible. However, FRACTURE also offers the advantage of assessing high visibility in the sagittal plane. When assessing cortical delineation in cases of biceps brachii tenosynovitis-related intertubercular groove sclerosis (16), FRACTURE and the transverse plane of PDW are considered adequate for evaluation.

The greater tubercle, where the infraspinatus and supraspinatus tendons attach, is visualized clearly on CT, FRACTURE, and PDW. Both CT and FRACTURE consistently achieved scores of 3 or higher, regardless of the imaging planes. Isolated fractures of the greater tuberosity (17), which can be quite small, pose challenges for diagnosis on radiographs; therefore, CT is required for the identification and classification of the fracture. Even though FRACTURE was only applied to normal dogs in this study, FRACTURE can offer easier fracture detection than CT, which was demonstrated in previous human studies to depict non-displaced avulsion fractures (11).

The lesser tubercle of the humerus and the site of insertion for the subscapularis tendon were easily delineated in CT, FRACTURE, and PDW. It was confirmed that both FRACTURE and PDW were found to be non-inferior when compared to CT through statistical analysis; however, FRACTURE generally provided clearer visualization compared to PDW. While the lesser tubercle is indirectly involved in shoulder stability, only a few cases of the pathologic condition of the lesser tuberosity have been reported in dogs (18–20). In three dogs with a large fragment in a fracture of the lesser tubercle, the fracture was diagnosed using radiographs (18–20). In the case of microfracture not observed in radiographs, FRACTURE could be considered for evaluating the lesser tubercle complementary to CT and PDW.

In humans, trabecular bone is generally evaluated for bone disorders such as osteoporosis and bone tumors because the trabecular bone is the supportive tissue in the medullar bone using trabecular bone scores in high-resolution CT and MRI (21, 22). In veterinary medicine, the assessment of the trabecular bone is required in many shoulder diseases, such as subchondral bone lesions, fractures, osteoarthritis, and OCD (23–26). In the trabecular compartment, 20% of the volume comprises bone, with the remaining space filled by bone marrow and fat (27). These inherent characteristics can lead to variations in the visualization and differentiation of trabecular bone between MRI and CT scans. In the field of veterinary medicine, when assessing the subchondral region, both CT and MRI sequences, such as T2W or PDW sequences, can be valuable (28). CT provides detailed information regarding bone structures, including subchondral bone defects or cysts. In contrast, T2W and PDW sequences in MRI can highlight features such as inflammation, edema, and hemorrhage, which may appear hyperintense (27, 28). However, in this study, as we compared normal subjects, hypersignal was not observed in PDW and T2W sequences. FRACTURE provided a clear visualization of the trabecular bone compared to other conventional MRI sequences. FRACTURE provided CT-like images that show high conspicuity of the trabecular bone similar to CT, although beam-hardening artifacts caused by the position of the forelimb reduced the conspicuity of the trabecular bone in CT images. In one normal dog, trabecular bone was not discernible in FRACTURE, likely due to fatty deposition leading to trabecular bone structure deterioration (29). MRI creates images based on the proton density of substances, causing materials with a high proton density, such as water, to appear bright and those with a low proton density, such as fat, to appear dark. However, when fat deposits in trabecular bone, the density contrast between fat and bone decreases, leading to an indistinct trabecular pattern. This makes it challenging to visually distinguish trabecular bones. There is potential in FRACTURE to replace CT for evaluating the trabecular bones by excluding obese dogs more prone to fat deposition.

The shoulder joint was visualized in all sequences and all dogs (except for T1W), specifically in CT, FRACTURE, and PDW. This observation aligns with a previous study (30) that emphasized the usefulness of the PDW sequence for assessing joint effusion and pathologic fluid accumulation within the joint. It is considered that the boundary between the glenoid cavity and the head of the humerus, as well as components of the shoulder joint, can be evaluated using FRACTURE and PDW as alternatives to CT.

Motion artifacts mildly affect the shoulder on MRI, and breath-hold and artifact reduction techniques (31) have been suggested to reduce them in humans. In the case of FRACTURE, which involves the magnitude summation of multiple echoes, it is considered more susceptible to motion artifacts due to respiration compared to conventional MRI. While multiple beam-hardening artifacts occurred in CT, they had minimal impact on image evaluation. Three dogs were extended to 140° or more during image acquisition, resulting in reduced beam-hardening artifacts compared to other images. Extending the forelimb as much as possible is considered to reduce beam-hardening artifacts by aligning the angle of the humerus and helical CT as closely as possible to 90°. Although significant artifacts were observed in CT and FRACTURE, these artifacts did not substantially affect image assessment. The cause of the subtle pixelated appearance observed in a dog’s T1W image remains unknown. Nevertheless, the image quality did not pose significant issues for image evaluation.

This study applied an innovative MRI technique called FRACTURE to animals, which offers superior cortical and trabecular bone contrast in comparison to conventional MRI sequences. FRACTURE is based on a gradient echo sequence with a subtraction post-processing step; however, this approach comes with certain inherent challenges. Sensitivity to B0 inhomogeneities and susceptibility artifacts, leading to imprecise signal localization, can be problematic, especially when orthopedic hardware is present (11). Additionally, the subtraction technique may highlight areas besides bone, such as air, that appear hyperintense on images; however, they are easily distinguishable using conventional MRI sequences (11).

All MR images in this study were obtained using 3D acquisition mode for the FRACTURE sequence as well as conventional T1W, T2W, and PDW sequences. The shoulder joint in healthy beagle dogs exhibited excellent visualization on FRACTURE and T1W, T2W, and PDW sequences using 3D TSE. Typically, the muscles and bones in the shoulder joint are assessed using 2D T2W, T1W, and PDW sequences. Nevertheless, when utilizing 3D TSE for the shoulder joint, it reduces MRI examination time without any loss of information, and perfect concordance with the 2D sequence can be achieved (32). While this study did not directly compare 2D and 3D imaging, it confirmed the excellent visibility of the four major tendons (biceps brachii, supraspinatus, infraspinatus, and subscapularis tendons) in all planes. This finding suggests that using 3D FSE offers a broader evaluation plane and increased visibility of the shoulder joint compared to 2D imaging.

This study has some limitations. First, there were variations in the imaging positions between CT and MRI. This discrepancy arises from the fact that attempting to capture images in the same lateral recumbency position in the CT scans as in an MRI led to severe artifacts on the CT images. However, although the positioning was not identical, efforts were made to extend both forelimbs maximally, ensuring that images were acquired within a comparable angular range for both modalities. Consequently, the impact on the assessment of the shoulder joint was minimized to the greatest extent possible. Second, this study was performed only in a small number of clinically healthy dogs and did not include a comparative evaluation of direct joint lesions and imaging characteristics that arise when accompanied by surrounding soft tissue lesions. Further studies, including a large population of dogs with pathologic conditions affecting the shoulder joint, are needed.

The current study demonstrates the feasibility of the FRACTURE sequence for visualizing the normal shoulder joint in healthy beagle dogs. FRACTURE showed high grades of cortical delineation and trabecular bone conspicuity of the shoulder joint, comparable to those seen in CT in dogs. These findings indicate that the FRACTURE sequence can complement conventional MR sequences in evaluating the shoulder joint by providing detailed assessments of cortical and trabecular bones. Using a FRACTURE sequence can reduce anesthesia time and costs by eliminating the need for an additional CT scan to evaluate the osseous part of the shoulder joint in dogs.

## Data availability statement

The original contributions presented in the study are included in the article/supplementary material, further inquiries can be directed to the corresponding author.

## Ethics statement

The animal study was approved by Institutional Animal Care and Use Committee at Seoul National University. The study was conducted in accordance with the local legislation and institutional requirements.

## Author contributions

SR: Conceptualization, Data curation, Formal analysis, Investigation, Methodology, Writing – original draft. SP: Formal analysis, Writing – original draft. EK: Writing – original draft, Methodology. HW: Methodology, Writing – original draft. C-yJ: Methodology, Writing – original draft. JY: Writing – review & editing. JC: Writing – review & editing, Conceptualization, Funding acquisition, Project administration, Supervision, Validation.
